# Oxidative Stress in Age-Related Macular Degeneration: Nrf2 as Therapeutic Target

**DOI:** 10.3389/fphar.2018.01280

**Published:** 2018-11-05

**Authors:** Ilaria Bellezza

**Affiliations:** Department of Experimental Medicine, University of Perugia, Perugia, Italy

**Keywords:** oxidative stress, light-induced photooxidative damage, cigarette smoke, aging, Nrf2 activators

## Abstract

Age-related macular degeneration is one of the leading causes of vision loss in the elderly. Genetics, environmental insults, and age-related issues are risk factors for the development of the disease. All these risk factors are linked to the induction of oxidative stress. In young subjects retinal pigment epithelial cells mitigate reactive oxygen generation by the elimination of dysfunctional mitochondria, via mitophagy, and by increasing antioxidant defenses via Nrf2 activation. The high amount of UV light absorbed by the retina, together with cigarette smoking, cooperate with the aging process to increase the amount of reactive oxygen species generated by retinal pigment epithelium where oxidative stress arises. Moreover, in the elderly both the mitophagic process and Nrf2 activation are impaired thus causing retinal cell death. This review will focus on the impact of oxidative stress on the pathogenesis of age-related macular degeneration and analyze the natural and synthetic Nrf2-activating compounds that have been tested as potential therapeutic agents for the disease.

## Introduction

The retina is a multilayered sensory structure that lines the inner surface of the back of the globe of the eye. The macula is a well-defined region of the retina with an approximate size of 0.6 mm devoted to the “high definition vision.” It can be subdivided into two zones: a central zone, the fovea, and a peripheral zone, the parafovea. The fovea contains a high percentage of cones, the photoreceptor cells devoted to the photopic vision, i.e., vision under well lit conditions; the parafovea is rich in rods, the photoreceptor cells devoted to night vision. The interplay between the macula’s cells guarantees central vision acuity which is indispensable for the most common daily activities (Datta et al., 2017).

The external layer of the retina is the retinal pigment epithelium (RPE) composed of highly specialized, polarized epithelial cells whose apical side is in contact with photoreceptor outer segments and basal side is in contact with Burch’s membrane, the internal layer of the choroid. RPE is important for the health of phototeceptor cells. Indeed, RPE cells phagocytose photoreceptor outer segments daily to guarantee their optimal functionality. Moreover, RPE cells transport metabolic waste through Burch’s membrane to the choroid to be eliminated (Kevany and Palczewski, 2010).

Age related macular degeneration (AMD), the principal cause of blindness in western countries (Congdon et al., 2004), is caused by the loss of RPE cells and photoreceptors in large zones of the macula. AMD is characterized by the presence of extracellular depositions, known as drusen accumulating between Burch’s membrane and the RPE. Advanced glycation end-products (AGEs) and carboxyethylpyrrole adducts (produced by the oxidative modification of fatty acids in photoreceptor tips) have been found in drusen isolated from AMD samples. The presence of these molecules, related to oxidative damage (Farboud et al., 1999; Crabb et al., 2002), underpins the concept that oxidative stress plays a major role in AMD pathogenesis and progression (Beatty et al., 2000). It is worth noting that AGEs can be recognized by receptor for advanced glycation endproducts (RAGE), a transmembrane receptor that exerts pro-inflammatory functions through nuclear factor-κB (NF-κB) signaling (Lin, 2006), thus implicating inflammation as another pathogenic causes of AMD.

Based on these premises, this review describes how oxidative stress contributes to macular degeneration and the effects of pharmacologically induced antioxidant defenses.

## Oxidative Stress and AMD

During cellular metabolism living organisms produce reactive oxygen species (ROS) from molecular oxygen. The major site of ROS production is the mitochondrial electron transport chain where some electrons leak from the transport process and spontaneously react with molecular oxygen, producing superoxide anion. Other enzymes, such as NADPH oxidase and xanthine oxidase contribute to ROS generation (Birben et al., 2012).

Reactive oxygen species levels are strictly regulated to maintain cellular homeostasis. Oxidative stress (OS) refers to a condition in which ROS levels accumulate to the extent that cellular macromolecules are damaged and apoptosis ensues (Birben et al., 2012). On the other hand, reductive stress is the name given to the condition when ROS levels are too low, such that the normal functions of the cell are affected. In homeostatic conditions, ROS are maintained at levels that support the normal cell functioning and guarantee redox signaling (Bellezza et al., 2018). The correct levels of ROS are underwritten by the antioxidant system, which is compromised of enzymes and non-enzymatic molecules. Non-enzymatic antioxidants include low-molecular-weight compounds, i.e., vitamins C and E, β-carotene, and glutathione. The majority of the enzymatic antioxidant defenses, i.e., SODs, catalase, and the enzymes responsible for glutathione metabolism, are regulated at transcriptional levels by the transcription factor nuclear factor erythroid 2-related factor 2 (Nrf2) (Bellezza et al., 2010) (see below).

The main risk factors for the development of AMD are aging, ethnicity, genetics and environmental insults, including cigarette smoking, high fat diet and light-induced photooxidative reactions (Organisciak and Vaughan, 2010; Datta et al., 2017). Aging, cigarette smoking and photo-oxidative reactions share the capacity to increase in ROS generation and promote OS.

### Light-Induced Oxidative Stress

Light is an electromagnetic radiation which can be translated into a visual information by complex interactions between the eye and the brain. Only a portion of the electromagnetic spectrum interacts with the eye and includes wavelengths from ultraviolet (100–400 nm) to infrared (above 760 nm) (Ivanov et al., 2018).

As long ago as 1966 Noell and co-workers theorized light damage hypotheses, including the occurrence of light-induced oxidative reactions. In particular, ultraviolet and blue light are considered responsible for the retinal damage associated with AMD (Noell et al., 1966; Chalam et al., 2011). It is predicted that increasing exposure to video displays and the use of light-emitting diodes (LEDs) as light sources will increase the contribution of blue light-induced phototoxicity to human retinal diseases (Jaadane et al., 2015). Photo-oxidative damage occurs when light interacts with an endogenous chromophore in the ocular tissue including visual pigments, proteins, flavoproteins and the naturally occurring pigment granules of melanin and lipofuscin in the RPE (Ivanov et al., 2018). The absorption of light by chromophores causes their excitation to a triplet state that, being a highly reactive, rapidly interact with other molecules, including molecular oxygen thus leading to generation of ROS (Chalam et al., 2011; Ivanov et al., 2018). Therefore, OS has been identified as one of the major players in light-induced cellular stress.

Exposure to ultraviolet radiation induces degeneration of RPE mitochondria, known to increase ROS generation, accompanied by a reduction in ATP generation. Since one of the major function of RPE is the phagocytosis of photoreceptor outer segments after photoactivation, a decrease in ATP generation might be responsible for a reduction in RPE phagocytic ability that culminates in RPE hyperpigmentation, a risk factor for AMD (Chalam et al., 2011). Moreover, ultraviolet radiation drives the upregulation of pro-inflammatory molecules through NF-κB activation, a condition that may accelerate drusen formation (Chalam et al., 2011).

### Oxidative Stress and Aging

The aging process is defined as the sum of deteriorative alterations that decreases both the fitness of the organism and the ability to maintain homeostasis (Finch and Ruvkun, 2001). The “free radical theory” of aging, proposed in 1956 by Harman (Harman, 1956), states that the accumulation of free radicals during lifespan leads to the accrual of oxidative damage to various classes of macromolecules that, in the end, is responsible for the decline in the physiological fitness of the organism. This was followed by the “oxidative stress theory” of aging stating that the aging process is driven by an imbalance between pro-oxidant species and antioxidant defenses (Sies and Cadenas, 1985). It is important to note, however, that ROS are not only hazardous molecules causing OS, but have a fundamental role in cellular signaling, namely redox signaling, that ensure correct cellular functions. The concept of reductive stress, defined as a condition of sustained increase in cellular reducing equivalents, associated with excessive Nrf2 activation, has recently come to the attention of the scientific community (Bellezza et al., 2018). For these reasons the “redox stress hypothesis” of aging has recently been proposed and states that aging-associated functional declines is primarily driven by a progressive disruption of the redox-regulated signaling mechanisms (Sohal and Orr, 2012).

An increase in oxidative modifications of macromolecules and a concomitant decrease in the antioxidant defenses are associated to the aging process (excellently reviewed in Sohal and Orr, 2012; Jacob et al., 2013; Ewald, 2018). The leakage of electrons from the electron transport chain might increase with age, explaining the age-related increase in ROS generation (Cadenas and Davies, 2000; Sohal and Orr, 2012). In addition, a diminished antioxidant capacity and an impaired adaptive induction of antioxidants has been observed during the aging process (Zhang et al., 2015).

Retinal pigment epithelium, having a high metabolic activity, possesses an elevated number of mitochondria (Datta et al., 2017) to generate enough ATP to accomplish all its physiological functions. Therefore, the age-related mitochondrial malfunctioning can increase OS in the RPE thus leading to AMD (Golestaneh et al., 2016).

Aging is also associated with a chronic low-grade inflammation, known as inflammageing, a condition characterized by elevated levels of inflammatory markers that provides high susceptibility to morbidity, including AMD (Zhuang and Lyga, 2014).

Also a role for cellular senescence of the RPE in the etiology of AMD has been proposed (Kozlowski, 2012). Cell senescence, i.e., the state of permanent cellular division arrest, has been involved in aging and in age-related diseases. Senescent cells has been found in eye diseases such as cataracts, and glaucoma (Naylor et al., 2013). Mitochondrial ROS have a causative role in cellular senescence and exposure to pro-oxidants induce the senescence process in proliferating human RPE *in vitro* (Blasiak et al., 2017).

The eye contains a circadian system and aging affects the circadian rhythm of the retina (Baba and Tosini, 2018). As an example, melatonin through melatonin receptors, regulates the daily rhythm of photoreceptor phagocytosis and melatonine receptors knock-out mice showed lipofuscin accumulation in the RPE (Laurent et al., 2017). These results suggest that alterations in the circadian rhythm can be involved in AMD pathogenesis, but more research in this area is warranted. Circadian clock regulates the expression of half of the mammalian protein which, in turn, are involved in drug transport/metabolism or are drug targets themselves (Ruben et al., 2018), thus circadian rhythm can be considered as target for AMD therapy.

## Cigarette Smoking and Oxidative Stress

Cigarette smoke is a strong oxidant composed of approximately 4700 chemical components including ROS, epoxides, peroxides, nitric oxide, peroxynitrite (Rahman and MacNee, 1996). Although cigarette smoking is one of the principal non-genetic factors associated with AMD pathogenesis, a direct damage of the RPE cells by cigarette smoke has been demonstrated only in 2008 by Fujihara and co-workers (Fujihara et al., 2008; Cano et al., 2010). Mice exposed to 6 months of cigarette smoke in a chamber that produces emphysema with evidence of oxidative damage, also develop RPE apoptosis and basal drusen-like deposits. Moreover, cigarette smoke extract mediates autophagy-impairment in RPE cells and affect cell viability by inducing ROS generation (Govindaraju et al., 2017). Cigarette smoke extract and one of its components, 2-ethylpyridine, enhance mitochondrial fragmentation and dysfunction (Mansoor et al., 2014; Huang et al., 2015) suggesting a potential role for cigarette smoking in the reduced phagocytic capacity associated with AMD.

Moreover, exposure to cigarette smoke results in production and release of pro-inflammatory molecules by immune cells via the activation of the NF-κB pathway (Rom et al., 2013; Marinucci et al., 2018).

## Antioxidant Defenses

Retinal pigment epithelium redox homeostasis relies on the activation of the transcription factor Nrf2. Under basal conditions, Nrf2 activity is maintained at low levels by the binding to its inhibitor Kelch ECH-associated protein 1 (Keap1), a Cul3-based E3 ligase that polyubiquitinates Nrf2 leading to its constitutive degradation by the proteasome. The low basal Nrf2 activity allows the maintenance of redox homeostasis. Under OS conditions, two redox-sensitive cysteine residues of Keap1 become oxidized resulting in the inhibition of its ubiquitin ligase activity. As a consequence, newly synthesized Nrf2 is not degraded, and translocates to the nucleus where it binds to the antioxidant and electrophilic responsive element (ARE/EpRE) sequences on the regulatory region of target genes leading to the induction of the antioxidant response (Bellezza et al., 2018). Besides Keap1, other two ubiquitin ligase complexes can regulate Nrf2 activation, i.e., F-box/WD repeat-containing protein 1A (βTRCP) and synoviolin (HRD1) (Tebay et al., 2015; Rojo de la Vega et al., 2018), whose possible involvement in AMD is worthy of investigation. It is also to note that expression of Nrf2 can be controlled by the molecular clock protein, BMAL1, (Early et al., 2018).

Due to the high amount of ROS produced in the retina, the RPE has adapted to life under OS conditions (Handa, 2012). The presence of several chromophores in the retina can provide protection against light induced damage by absorbing excess light energy. In the aging RPE, an accumulation of melanofuscine granules, containing both melanin and lipofuscin, has been observed, and this phenomenon correlates with AMD development (Chalam et al., 2011). Moreover, as discussed above, mitochondria are the main source of ROS in the RPE and their leakage increases with aging. To maintain the correct cell functions, the RPE removes damaged or malfunctioning mitochondria through the process of mitophagy, a mitochondrial-specific type of autophagy. It has been hypothesized that mitophagy impairment may play a role in AMD pathogenesis (Hyttinen et al., 2018). The outer mitochondrial membrane proteins B-cell leukemia/lymphoma 2 (BCL-2)/adenovirus E1B interacting protein 3 (BNIP3) and Nip-like protein X (NIX) cause an increase in ROS generation which, in turn, induces mitochondrial depolarization, autophagy and mitophagy (Vande Velde et al., 2000; Ding et al., 2010; Ney, 2015). However, a role for BNIP3 and NIX in AMD is still to be elucidated.

The accumulation of oxidatively damaged molecules found in AMD suggests that the antioxidant defense cannot cope with the increasing amount of ROS (Datta et al., 2017). Mounting evidence suggests that aging induces a decline in the antioxidant capacity via a reduction in Nrf2 signaling (Sachdeva et al., 2014; Zhang et al., 2015). Nrf2 activation has been linked to mitochondrial structural and functional integrity where its role is of particular importance under stressful conditions (Dinkova-Kostova and Abramov, 2015). Furthermore, the known cross-talk between Nrf2 and NF-κB implicates that a decline in Nrf2 signaling in exacerbated NF-κB activation further increasing inflammation (Bellezza et al., 2010, 2018; Wardyn et al., 2015).

## Nrf2 Activating Compounds in AMD Therapy

Awareness of the importance of Nrf2 in retinal disease came from the finding that Nrf2-deficient mice develop ocular pathology similar to human AMD (Zhao et al., 2011).

Different types of stresses have been employed to induce OS in retinal epithelial cells such as UV exposure, hydrogen peroxide, or acrolein, a component of cigarette smoke. In these experimental settings several known or potential antioxidant compounds have been tested either *in vitro* or *in vivo* and a number of clinical trials have investigated the effects of antioxidants on AMD progression (Nakagami, 2016).

The first experimental evidence that Nrf2 activation can protect the RPE from photooxidative damage came in 2004 when Gao and Talalay demonstrated that sulforaphane, contained in broccoli and cabbages, protects human adult RPE cells from ultraviolet light-induced damage by increasing Nrf2-regulated glutathione levels and NAD(P)H:quinone oxidoreductase activity (Gao and Talalay, 2004). Sulforaphane protects mouse retina from ultraviolet light by upregulating the expression of thioredoxin, an antioxidant protein whose expression is driven by Nrf2. Moreover, sulforaphane exerted protection of ARPE-19 cell line exposed to 400 μM H_2_O_2_ by up-regulating the translation of thioredoxin and Nrf2 (Tanito et al., 2005) and has been suggested to promote regeneration of retinal cells (Dulull et al., 2018). Recently the protective effect of sulforaphane on RPE cells was correlated with preventing mitochondrial fission independently of Nrf2 activation (O’Mealey et al., 2017).

Curcumin, a natural compound found in *Curcuma longa*, protects ARPE-19 cells from up to 1mM H_2_O_2_ exposure by inducing the Nrf2 driven gene hemeoxygenase 1 (HO-1) (Mandal et al., 2009) and a curcumin analog, 1, 5-bis (2-trifluoromethylphenyl)-1, 4-pentadien-3-one, exerted protection against acrolein-induced oxidative damage by inducing Nrf2 (Li et al., 2013). Moreover, curcumin fed rats (0.2% for 2 weeks) were protected from light-induced retinal degeneration by down-regulating inflammatory genes via NF-κB inhibition (Mandal et al., 2009).

The carotenoids zeaxanthin and lutein preserve photoreceptors against light damage by mitigating OS (Yu et al., 2018). It has been shown that lutein activates Nrf2 in ARPE-19 cells (Frede et al., 2017) and that mesozeaxanthin protects against chronic and cumulative eye damage by reducing OS (Orhan et al., 2016). A prospective, randomized controlled study with 114 early AMD patients demonstrated that 25 g of Goji berries supplementation per day for 90d improves macular pigment optical density by increasing serum zaxantin levels (Li et al., 2018) and, as excellently reviewed by Buscemi and co-workers high lutein intake, either through diet or as nutritional supplement, has beneficial effects on AMD (Buscemi et al., 2018). Lutein and Zeaxantin can also reduce NF-κB activation in the retina (Tuzcu et al., 2017).

Carnosic acid from *Rosmarinus officinalis* and *Salvia officinalis*, salvianolic acids from Radix *Salvia miltiorrhiza*, mangostin from *Garcinia mangostana*, taxifolin a flavonol from conifers, were all effective against OS damages in retinal cells via activation of Nrf2 (Rezaie et al., 2012; Zhang et al., 2014; Liu et al., 2016b; Xie et al., 2017).

Also the Mediterranean diet, characterized by high consumption of plant foods, olive oil as primary fat source, and moderate consumption of wine (Willett et al., 1995) can be regarded as an Nrf2 activator (Martucci et al., 2017). A prospective cohort study of the Rotterdam Study I (RS-I) and the Antioxydants, Lipides Essentiels, Nutrition et Maladies Oculaires (Alienor) Study showed that higher adherence to the Mediterranean diet is associated with a reduced risk of advanced AMD (Merle et al., 2018). Furthermore, a nested case–control study within the Coimbra Eye Study demonstrated that high adherence to Mediterranean diet confers protection against the development of AMD (Raimundo et al., 2018).

The beneficial effects of Nrf2 activation on retinal cells has led to the synthesis of Nrf2 activators such as RS9 (Nakagami et al., 2015). RS9 decreases light-induced retinal cell death *in vivo* and *in vitro* (Inoue et al., 2017). Very recently it has been reported that RS9 protects RPE cells from sodium iodate-induced OS and adult zebrafish retina from light-induced damage by increasing Nrf2-dependent HO-1 expression (Saito et al., 2018). RTA 408, a synthetic triterpenoid able to activate Nrf2, protects cultured RPE cells from OS (Liu et al., 2016a). Investigation of other molecules that exert cytoprotection by activating Nrf2 and potentially by inhibiting NF-κB (Bellezza et al., 2014; Grottelli et al., 2016) are warranted to ascertain vision preservation in early AMDpatients.

## Conclusion

**FIGURE 1:**
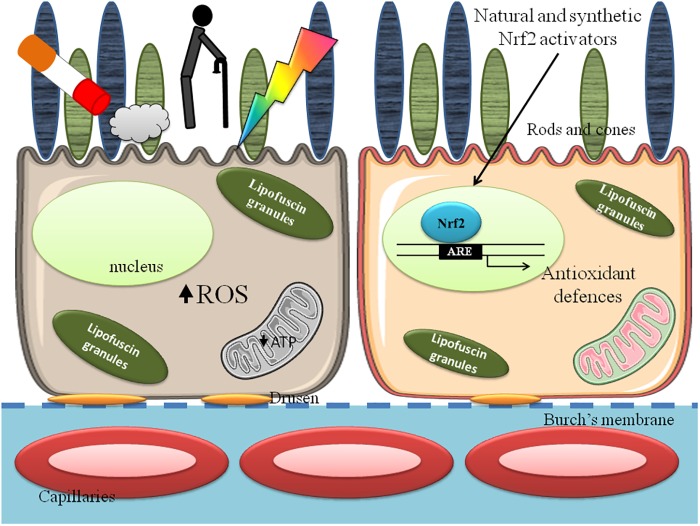
Retinal pigment epithelium (RPE) in age related macular degeneration (AMD). Cigarette smoke, aging and light absorption increase reactive oxygen species (ROS) formation and decrease mitochondrial function, lowering ATP synthesis and affect RPE cell functions **(Left)**. The exposure to Nrf2 activating compounds increases antioxidant defenses and ameliorate mitochondrial and cellular functions **(Right)**.

Oxidative stress is an important contributor of AMD, and Nrf2 activation exerts protective effects that can be enhanced by pharmacologic meaning (Figure 1).

An increase of the antioxidant defenses can provide novel and effective therapeutic strategies for this disease. However, it will be important to apply Nrf2 activators mindful of the concept of redox homeostasis, since there is a fine line between beneficial and potentially damaging effects of Nrf2 activation. However, adverse systemic effects during the treatment of ocular diseases might be minimized by local pharmacological intervention such as intravitreal injections or by the use of eye drops.

## Author Contributions

IB conceived the work, analyzed bibliogaphical data, and wrote the manuscript.

## Conflict of Interest Statement

The author declares that the research was conducted in the absence of any commercial or financial relationships that could be construed as a potential conflict of interest.
